# Dual nanocomposite carrier transport layers enhance the efficiency of planar perovskite photovoltaics†

**DOI:** 10.1039/c8ra01532e

**Published:** 2018-04-04

**Authors:** Hsi-Kuei Lin, Jia-Xing Li, Hao-Cheng Wang, Yu-Wei Su, Kaung-Hsiung Wu, Kung-Hwa Wei

**Affiliations:** Department of Materials Science and Engineering, National Chiao Tung University 300 Hsinchu Taiwan khwei@mail.nctu.edu.tw; Department of Electrophysics, National Chiao Tung University 300 Hsinchu Taiwan; Department of Chemical Engineering, Feng Chia University Taichung 40724 Taiwan

## Abstract

In photovoltaic devices, more effective transfer of dissociated electrons and holes from the active layer to the respective electrodes will result in higher fill factors and short-circuit current densities and, thus, enhanced power conversion efficiencies (PCEs). Planar perovskite photovoltaics feature an active layer that can provide a large exciton diffusion length, reaching several micrometers, but require efficient carrier transport layers for charge extraction. In this study, we employed two nanocomposite carrier transfer layers—an electron transport layer (ETL) comprising [6,6]phenyl-C_61_-butyric acid methyl ester (PC_61_BM) doped with the small molecule 4,7-diphenyl-1,10-phenanthroline (Bphen), to enhance the electron mobility, and a hole transfer layer (HTL) comprising poly(3,4-ethylenedioxythiophene):polystyrenesulfonate (PEDOT:PSS) doped with molybdenum disulfide (MoS_2_) nanosheets, to enhance the hole mobility. We used ultraviolet photoelectron spectroscopy to determine the energy levels of these composite ETLs and HTLs; atomic force microscopy and scanning electron microscopy to probe their surface structures; and transmission electron microscopy and synchrotron grazing-incidence small-angle X-ray scattering to decipher the structures of the ETLs. Adding a small amount (less than 1%) of Bphen allowed us to tune the energy levels of the ETL and decrease the size of the PC_61_BM clusters and, therefore, generate more PC_61_BM aggregation domains to provide more pathways for electron transport, leading to enhanced PCEs of the resulting perovskite devices. We used quantitative pump-probe data to resolve the carrier dynamics from the perovskite to the ETL and HTL, and observed a smaller possibility of carrier recombination and a shorter injection lifetime in the perovskite solar cell doubly modified with carrier transport layers, resulting in an enhancement of the PCE. The PCE reached 16% for a planar inverted perovskite device featuring an ETL incorporating 0.5 wt% Bphen within PC_61_BM and 0.1 wt% MoS_2_ within PEDOT:PSS; this PCE is more than 50% higher than the value of 10.2% for the corresponding control device.

## Abstract 2

In photovoltaic devices, more effective transfer of dissociated electrons and holes from the active layer to the respective electrodes will result in higher fill factors and short-circuit current densities and, thus, enhanced power conversion efficiencies (PCEs).

## Graphical abstract

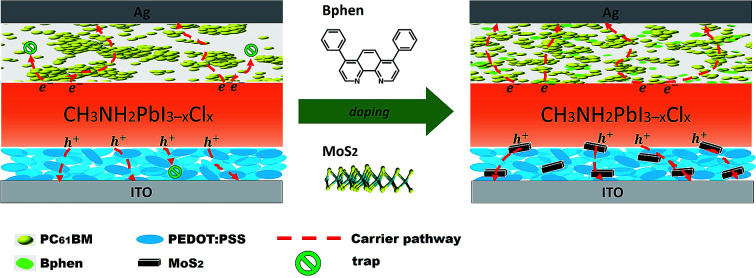

## Introduction

Organometal halide perovskite photovoltaics have attracted much attention in recent years. Perovskite is an excellent photovoltaic active layer (one that absorbs light energy and converts it into electric current) because of its panchromatic light absorption, ambipolar transport, and very long electron/hole diffusion lengths. Furthermore, perovskite photovoltaics are less expensive than traditional silicon-based photovoltaics. The manufacture of a perovskite photovoltaic is simple and convenient—particularly in the case of planar structures that do not require processing at high temperature or pressure. The highest power conversion efficiencies (PCEs) measured for perovskite photovoltaics increased rapidly over of the recent years as such, perovskite is now considered the photovoltaic material having the most potential.^1–3^

Based on their layered structures that determine the direction of flow of the electrons and holes, perovskite photovoltaics are divided into two types: conventional and inverted.^4,5^ Inverted perovskite photovoltaics appear better suited to commercial requirements, and many researchers have been interested in developing suitable active layers, buffer layers, and electrodes.^6,7^ Although the perovskite layer in a device can display outstanding internal quantum efficiency, improving the contact between the perovskite layer and the carrier transport layers will ensure that the carriers move through the interfaces without being subjected to substantial losses, thereby realizing higher PCEs.^8–11^

[6,6]Phenyl-C_61_-butyric acid methyl ester (PC_61_BM) is commonly used as the electron transfer layer (ETL) in inverted planar perovskite photovoltaics.^12–14^ PC_61_BM films prepared through spin-coating can feature film defects when not modified, resulting in a higher probability of electron–hole recombination at the perovskite–cathode interface.^15^ Bilayer structures, such as those formed from 4,7-diphenyl-1,10-phenanthroline (Bphen) and PC_61_BM, have been adopted to form structural ETLs.^16^ Although the use of Bphen can provide good performance, most research has focused on improving the contact between the ETL and the electrode. In a previous study, a small amount of nanostructured polystyrene-*block*-poly(ethylene oxide) (PS-*b*-PEO) copolymer was incorporated with PC_61_BM to optimize the morphology—forming smaller clusters that aggregated into domains—and enhance the PCE.^17,18^ The relatively high electron mobility of Bphen ensures that it does not hamper the electron mobility in the ETL when incorporated into PC_61_BM^19^.

Two-dimensional (2-D) monolayers of semiconducting transition metal dichalcogenides (TMDs) have direct band gaps and possess optical properties suitable for optoelectronic applications in light-emitting diodes^20^ and photovoltaics.^21^ Molybdenum disulfide (MoS_2_) is one of the most notable examples of a single-layer TMD; it has attracted great attention for applications in solar cell devices because of its interesting semiconducting and photonic properties.^22^ Similar to graphene, when MoS_2_ is converted from a bulk structure to a single-layer structure, its material properties undergo a significant change. Recently, Capasso *et al.* reported MoS_2_ flakes act as an active buffer layer between the perovskite and the spiro-OMeTAD in the conventional perovskite solar cells structure and as a protective layer for improving stability of perovskite solar cells, compared with those having spiro-OMeTAD alone.^23^ MoS_2_ can decrease iodine migration from the perovskite layer to the HTL, resulting in an improvement of the PCE and stability, and the formation of ITO pathways from the metal electrode to the perovskite layer. Although MoS_2_ nanosheets are potentially good hole-transporting layers, the work function of MoS_2_ has a mismatched band structure when working as the hole transfer layer (HTL) for perovskite active layer.^24,25^ We have used UV-ozone-treated MoS_2_ to provide a high carrier mobility that is suitable for application in HTLs; such treatment modifies the work function of the MoS_2_ layer.^26–28^

In this present study, we adopted two nanocomposites—an ETL featuring the organic molecule Bphen incorporated into PC_61_BM and an HTL featuring MoS_2_ nanosheets incorporated into poly(3,4-ethylenedioxythiophene):polystyrenesulfonate (PEDOT:PSS)—to enhance carrier transport from the perovskite to the electrodes. We analyzed the morphologies and properties of the composite ETL and HTL. Because Bphen has relatively high electron mobility, when incorporated in PC_61_BM we could directly modify the contacts to improve the devices' PCE performance. Because of the excellent electrical properties of MoS_2_, PEDOT:PSS incorporating MoS_2_ results in effective carrier transfer to the electrode. We expected that the presence of Bphen—which interacts with fullerenes to some extent—would shrink the size of the PC_61_BM clusters, resulting in more PC_61_BM clusters (based on mass balance) and, therefore, provide more pathways for efficient electron transfer to the electrode. Furthermore, we use ultrafast optical pump-probe spectroscopy to probe carrier recombination and injection from excitons to the perovskite boundary and to the ETL and HTL.^29,30^ We have probed the resulting electron and hole mobilities, photoluminescence (PL) spectra, and photovoltaic performance. Ultraviolet photoelectron spectroscopy (UPS) and morphological studies provided insight into the different properties obtained when incorporating Bphen and MoS_2_. We used grazing-incidence small-angle X-ray scattering (GISAXS) to determine the change in the PC_61_BM clusters.^31^ Finally, we performed transient absorption spectroscopy to understand how the carrier dynamics directly correlated to the efficiency of charge transport in the solar cell devices.

## Experimental

### Materials

ITO-coated glass substrates (Merck) were patterned using 2 M HCl, cleaned through ultrasonication (20 min each) with detergent, de-ionized water, acetone, and isopropyl alcohol, and then dried in an oven for 1 h. The materials for the HTLs and ETLs were PEDOT:PSS (CleviosTM P VP AI 4083) and PC_61_BM (FEM Technology), respectively. The perovskite active layers were made from the precursor I201 (Ossila) containing MAI : PbI_2_ : PbCl_2_ at 40 wt% (stoichiometry of 4 : 1 : 1) in anhydrous *N*,*N*-dimethylformamide (DMF). The small-molecule additive Bphen was obtained from Sigma-Aldrich; monolayer MoS_2_ powder was purchased from Ossila. The solutions for the ETL were prepared by dissolving PC_61_BM (20 mg mL^−1^) and Bphen at certain ratios in chlorobenzene, then stirring continuously in a N_2_-filled glove box for 12 h at 85 °C prior use. The solutions for the HTL [MoS_2_ (1 mg) in PEDOT:PSS solution (1 mL)] were dispersed through ultrasonic oscillation for 1.5 h.

### Device fabrication

The patterned ITO glass substrates were treated with UV ozone for 15 min prior to use; the HTL solution was then spin-coated (4000 rpm, 40 s) onto the ITO substrates. The ITO/HTL films were baked at 150 °C for 15 min under the atmosphere. The HTLs containing MoS_2_ were treated with UV ozone for 20 min before the films were transferred to a N_2_-filled glove box. The perovskite ink was heated at 70 °C for 1 h, cooled to room temperature, and deposited through spin-coating (4000 rpm, 30 s) onto the ITO/HTL surface. The films were then annealed at 90 °C for 50 min. The solutions for the ETL were passed through a PTFE filter (0.2 μm) and then spin-coated (1200 rpm, 30 s) onto the ITO/HTL/CH_3_NH_3_PbI_3−*x*_Cl_*x*_ surfaces. Device fabrication was completed through thermal evaporation of a 100 nm-thick film of Ag as the cathode under high vacuum (pressure: *ca.* 5 × 10^−7^ mbar). During the thermal evaporation process, a shadow mask was used to define a device area of 0.1 cm^2^.

### Device characterization

Current density–voltage (*J*–*V*) characteristics were recorded using a Keithley 2400 source-measure unit. A solar simulator, comprising a Xe lamp-based 150 W solar simulator (Newport 66902) and an AM 1.5G filter, was used to give an irradiance of 100 mW cm^−2^ on the surface of the solar cell. A calibrated mono-silicon diode equipped with a KG-5 filter, exhibiting a response in the range 300–800 nm, was used as a reference. External quantum efficiency (EQE) data were obtained using an EQE-D-3011 system (Enlitech, Taiwan) and a calibrated mono-silicon diode as a reference (displaying a response from 350 to 800 nm). Sample films were prepared by spin-coating the ETL and HTL solutions onto either 4 cm^2^ quartz (for UV-Vis spectroscopy) or a 2.25 cm^2^ silicon wafer (for UPS); PEDOT:PSS/CH_3_NH_3_PbI_3−*x*_Cl_*x*_/PC_61_BM:Bphen structures on a silicon wafer were prepared for GISAXS analyses. UV-Vis absorbance spectra were recorded using a Hitachi U-4100 spectrophotometer equipped with an integrating sphere, steady-state PL spectra were recorded in air using a Hitachi F-7000 fluorescence spectrophotometer; time-resolved PL spectra were collected in a customized single photon counting system which contains a sub-nanosecond pulsed diode laser (*λ* = 320 nm, PicoQuant, PLS 320), a grating spectrometer and a high-speed photomultiplier tube with the single photon counting card. Film morphologies were recorded using an atomic force microscope (Veeco Innova) operated in tapping mode. A JEOL-2010 transmission electron microscope was used to record images at a beam energy of 200 keV. UPS was performed at a sample bias of 4 V by He irradiation. Synchrotron GISAXS analysis [X-ray beam energy: 10 keV (*λ* = 1.24 Å); incident angle: 0.15°] was performed at the BL23A SWAXS beam line in the NSRRC, Hsinchu, Taiwan.

### Space Charge Limited Conduction (SCLC) mobility measurement

Electron- and hole-only devices were prepared having the structures ITO/ZnO/ETL(PC_61_BM:Bphen)/Ag and ITO/HTL(PEDOT:PSS:MoS_2_)/Ag, respectively. The charge carrier mobility was determined using the single carrier SCLC model, as described by the Mott–Gurney law,
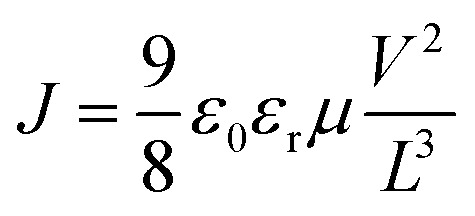
where *J* is the current density, *ε*_0_*ε*_r_ is the dielectric permittivity of the ETL or HTL; *L* is the thickness of the ETL or HTL; *μ* is the zero-field mobility; and *V* is the internal voltage in the device, given by*V* = *V*_appl_ − *V*_rs_ − *V*_bi_where *V*_appl_ is the applied voltage, *V*_rs_ is the voltage drop resulting from the relative difference in work function, and *V*_bi_ is the built-in voltage resulting from the relative difference in work function between the two electrodes.

### Transient absorption measurements

Time-resolved pump-probe studies were performed using a femtosecond Ti:sapphire laser system (Legend-USP-HP, Coherent) delivering a near-infrared (NIR) pulse (duration: *ca.* 40 fs; repetition rate: 5 kHz; center wavelength: 800 nm). The NIR laser pulse was split into two NIR pulses, with a power ratio of 10 : 1, using a beam splitter. The higher-intensity NIR pulse was focused into a β-barium borate crystal for second harmonic (SH) generation; the generated SH laser pulse was guided to a delay stage for retro reflection, and then focused onto the sample as a pump pulse. The lower-intensity NIR pulse (pulse energy: 5 μJ) was focused onto a sapphire plate (thickness: 2 mm) to generate a white light continuum (WLC); the WLC pulse was focused onto the sample as a WLC probe pulse. Using a parabolic mirror, both the pump (400 nm) and WLC probe (500–750 nm) pulses were focused onto the sample. A charge-coupled device camera (Series 2000, Entwicklungsburo Stresing), connected through an optical fiber and polychromator (CP140, Yobin Yvon), recorded the probe pulse transmitted through the sample. For measurements of the change in absorption with and without sample excitation, the pump frequency was modulated with an optical chopper running at 2.5 kHz. The difference absorption spectrum (Δ*A*) of the sample at each time delay between the pump and probe pulses was acquired using LabVIEW software. The delay was scanned using a delay stage inserted in the optical path of the pump pulses.

## Results and discussion

**Fig. 1:**
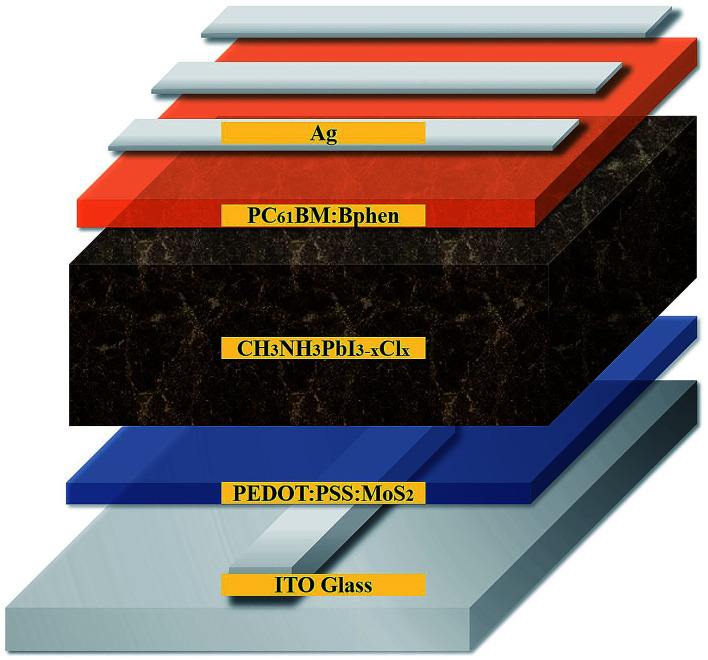
Schematic representation of the structure of the perovskite photovoltaics.

**Fig. 2:**
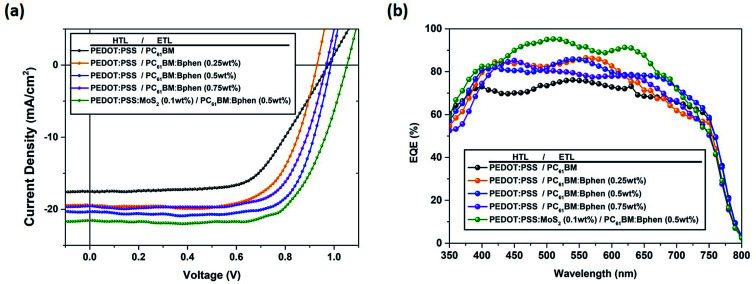
(a) *J*–*V* characteristics of perovskite devices incorporating Bphen at various contents (0, 0.25, 0.5, 0.75 wt%) in the ETL and incorporating MoS_2_ (0.1 wt%) in PEDOT:PSS as the HTL, based on ETL containing 0.5 wt% Bphen, measured under standard 1 sun AM 1.5G simulated solar irradiation. (b) EQE spectra of perovskite devices.

Fig. 1 provides a schematic representation of the planar structure of a perovskite photovoltaic device having the configuration indium tin oxide (ITO)/PEDOT:PSS:MoS_2_/CH_3_NH_2_PbI_3−*x*_Cl_*x*_/PC_61_BM:Bphen/Ag. Fig. 2(a) displays the *J*–*V* characteristics of planar perovskite solar cells incorporating pristine PC_61_BM and PC_61_BM:Bphen composites as ETLs and PEDOT:PSS:MoS_2_ composites as HTLs with perovskite CH_3_NH_2_PbI_3−*x*_Cl_*x*_ as the active layer. Table 1 presents the statistical data of the perovskite devices (15 devices), including their open-circuit voltages (*V*_oc_), short-circuit currents (*J*_sc_), fill factors (FFs), and PCEs. In the control device incorporating pristine PC_61_BM, the mean values of *V*_oc_ and *J*_sc_ and the FF and PCE were 0.99 V, 17.2 mA cm^−2^, 61.9%, and 10.2%, respectively (PCE_max_ = 10.6%). After incorporating Bphen at 0.25, 0.5, and 0.75 wt%, the values of *V*_oc_ decreased slightly to 0.96, 0.97, and 0.97 V, respectively. The devices in which the ETLs contained Bphen had values of *J*_sc_ higher those of devices prepared without Bphen. When we increased the amount of incorporated Bphen from 0.25 to 0.5 wt%, the short-circuit current density reached its highest value of 20.2 mA cm^−2^; it decreased to 17.9 mA cm^−2^ when Bphen was present at 0.75 wt%, possibly because self-aggregation of Bphen might have decreased the number of electron pathways. We used the ETL containing 0.5 wt% Bphen in PC_61_BM as the base case, and then incorporated MoS_2_ nanosheets into PEDOT:PSS as the HTL—forming dual-carrier transport layers. The presence of MoS_2_ affected the electronic properties of the HTL substantially, with the values of *V*_oc_ and *J*_sc_ increasing to 1.01 V and 21.3 mA cm^−2^, respectively. Fig. 2(b) displays corresponding EQE curves of the perovskite photovoltaic devices, revealing an enhancement in efficiency in the range from 400 to 750 nm in the EQE curve when incorporating both the Bphen/PC_61_BM ETL and the MoS_2_/PEDOT:PSS HTL. We recorded UV-Vis spectra to monitor the absorptions of the ETLs prepared with and without Bphen (Fig. S1, ESI†). We observed no apparent differences in the characteristic absorptions, suggesting that the enhanced values of *J*_sc_ arose from PC_61_BM aggregation and not from any additional ETL absorption. Although Bphen provides relatively good performance in terms of electron mobility, a decrease in the value of *J*_sc_ occurred when the ETL incorporated an excessive amount of Bphen, possibly the result of its aggregation. In the devices incorporating various concentrations of Bphen, the trend in the PCEs followed that of the FFs. The main reason for this improvement in PCE was that the FF increased significantly from 61.9% for the device prepared without Bphen to 66.3, 72.0, and 69.6% for the devices containing 0.25, 0.50, and 0.75 wt% Bphen, respectively. PC_61_BM films incorporating small amounts of Bphen can form more highly efficient ETL interfaces with the perovskite and the electrode, assisting in the extraction of electrons and decreasing the accumulation of charges at the interface.^32,33^ Furthermore, the MoS_2_/PEDOT:PSS HTL also enhanced the PCEs because the MoS_2_ nanosheets have excellent carrier mobility characteristics. Although the device FFs decreased slightly upon incorporation of the MoS_2_ nanosheets, the values of *V*_oc_ and *J*_sc_ increased sufficiently to compensate, resulting in the PCE increasing to 16% when incorporating an ETL containing 0.5 wt% Bphen in PC_61_BM. The higher values of *V*_oc_ and *J*_sc_ indicate that the presence of MoS_2_ decreases an energy loss during hole transfer.^34^ As a whole, when the ETL and HTL incorporated Bphen (0.5 wt%) and MoS_2_ (0.1 wt%), the PCE of the device reached its highest value of 16%—an increase of 56.9% relative to that of the control device.

**Table 1:** Device performance of perovskite solar cells

ETL	HTL	*V*_oc_ a [V]	*J*_sc_ b [mA cm^−2^]	FF^c^ [%]	*η* d (*η*_b_)^e^) [%]
PC_61_BM	PEDOT:PSS	0.99 ± 0.02	17.2 ± 0.6	61.9 ± 1.2	10.2 ± 0.3 (10.5)
PC_61_BM:Bphen (0.25 wt%)	PEDOT:PSS	0.96 ± 0.02	18.0 ± 1.0	66.3 ± 2.7	11.5 ± 0.4 (12.4)
PC_61_BM:Bphen (0.5 wt%)	PEDOT:PSS	0.97 ± 0.02	20.2 ± 0.6	72.0 ± 2.0	14.1 ± 0.6 (14.8)
PC_61_BM:Bphen (0.75 wt%)	PEDOT:PSS	0.97 ± 0.02	17.9 ± 1.4	69.6 ± 2.7	12.1 ± 0.8 (13.4)
PC_61_BM:Bphen (0.5 wt%)	PEDOT:PSS:MoS_2_ (0.1 wt%)	1.01 ± 0.03	21.3 ± 0.5	71.5 ± 2.2	15.4 ± 0.2 (16.0)

a *V*_oc_: open-circuit voltage.

b *J*_sc_: short-circuit current density.

c FF: fill factor.

d *η*: power conversion efficiency.

e *η*_b_: best power conversion efficiency.

Fig. S2(a) and (b)† present the UPS spectra recorded near the cutoff and onset energy regions. Based on the valence band energy level, *Φ*, we applied the formula*Φ* = *hv* − (*E*_1_ − *E*_2_)where *hv* was equal to 21.21 eV. The upper emission onset energies (*E*_1_) with high-energy-side shoulder tangents in the cutoff region and the lower emission onset energies (*E*_2_) of the secondary photoelectrons appeared in the valence band region.^35,36^ We used UPS spectra (photon source: He I, 21.21 eV) to examine the valence band energy levels of the ETL and HTL. Moreover, we recorded the transmission spectra of the ETLs to determine the optical band gap from the Tauc plot [(*αhv*)^2^*vs.* eV] to calculate the energy of the lowest unoccupied molecular orbital (LUMO), as displayed in Fig. S3;† here, *α* is equal to −ln(*T*/*t*), where *t* is the thickness of the ETL and *T* is the transmission.^37^

**Fig. 3:**
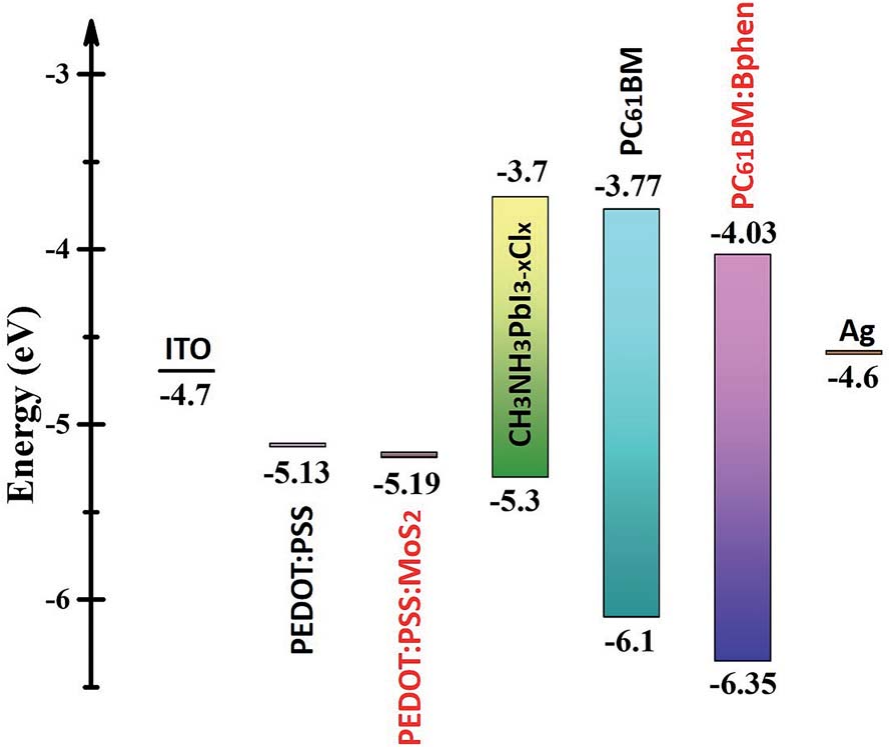
Energy level diagram of the materials investigated in this study.

Fig. 3 displays the energy level diagram of perovskite and the composite ETL (PC_61_BM:Bphen) and HTL (PEDOT:PSS:MoS_2_). The energy level of perovskite (CH_3_NH_2_PbI_3−*x*_Cl_*x*_) was taken from the literature.^38^ After incorporating Bphen, the LUMO energy level decreased from 3.77 to 4.03 eV. Because the LUMO of PC_61_BM:Bphen was lower than that of the pristine PC_61_BM, the devices incorporating the composite ETLs (involving Bphen) exhibited lower values of *V*_oc_. The potential difference between the perovskite and the ETL layer determined the strength of the built-in electric field in the interfaces. A higher built-in electric field accelerated electron transport to the cathode—thereby increasing the short-current density—but decreasing the open-circuit voltage (*V*_oc_). Although MoS_2_ is an n-type material, it can change into a p-type material after UV-ozone treatment.^25,28^ Therefore, after incorporating MoS_2_ into PEDOT:PSS, the energy level of the highest occupied molecular orbital (HOMO) decreased from 5.13 to 5.19 eV, resulting in the enhancement of the value of *V*_oc_ from 0.97 to 1.01 V. Fig. S4† presents X-ray photoelectron narrow-scan spectra (Mo 3d) of perovskite films prepared with and without UV-ozone treatment; we calculated the peaks area ratios of characteristic peaks: Mo^4+^ 3d_5/2_, Mo^4+^ 3d_3/2_, Mo^5+^ 3d_5/2_, Mo^5+^ 3d_3/2_, Mo^6+^ 3d_5/2_, Mo^6+^ 3d_3/2_ and S 2s. Mo4^+^ (Mo4^+^ 3d_5/2_ + Mo4^+^ 3d_3/2_), Mo5^+^ (Mo5^+^ 3d_5/2_ + Mo5^+^ 3d_3/2_) and Mo6^+^ (Mo6^+^ 3d_5/2_ + Mo6^+^ 3d_3/2_) representative MoS_2_, Mo_2_O_5_ and MoO_3_. Fig. S4(c)† shows Mo^5+^ areas (Mo^5+^ 3d_5/2_ + Mo^5+^ 3d_3/2_) and Mo^6+^ areas (Mo^5+^ 3d_5/2_ + Mo^5+^ 3d_3/2_) change from 29.2% and 25.5–17.3% and 38.1% after UV-ozone treatment.^39^ Mo_2_O_5_ (Mo^5+^) may be partially oxidized to MoO_3_ (Mo^6+^) by the oxygen atoms which can reduce the O vacancies.^40^ The oxygen atoms penetrated into the monolayer MoS_2_ after UV-ozone treatment, possibly partially filling the S vacancies and passivating the structural defects; as a result, the HOMO of the ETL could be tuned to a suitable energy level.^28^

**Fig. 4:**
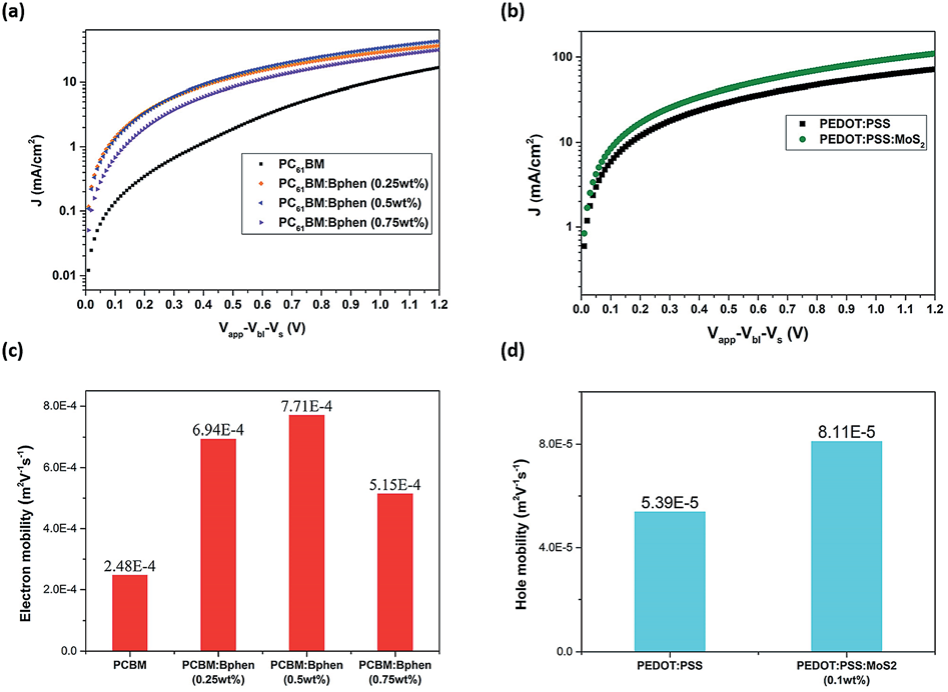
(a and b) current density–electric field log–log plots of (a) electron-only diodes (configuration: ITO/ZnO/PC_61_BM/Ca/Al) containing ETL films prepared with various amounts of Bphen and (b) hole-only diodes (configuration: ITO/PEDOT:PSS/MoO_3_/Ag) containing HTL films prepared with and without MoS_2_. (c) Electron mobility in PC_61_BM layers incorporating Bphen at various contents. (d) Hole mobility in PEDOT:PSS layers prepared with and without MoS_2_.

Fig. 4 presents current density–electric field plots for the electron- and hole-only diodes; we used the SCLC model to determine the carrier mobility of the devices. The electron mobility when Bphen was present in the ETL was higher than that of the neat PC_61_BM film. When Bphen was incorporated at 0.5 wt% in PC_61_BM, the electron mobility was optimal, increasing threefold from 2.48 × 10^−4^ m^2^ V^−1^ s^−1^ for the device prepared without Bphen to 7.71 × 10^−4^ m^2^ V^−1^ s^−1^. In addition, the hole mobility when MoS_2_ was present in the ETL also increased, from 5.39 × 10^−5^ to 8.11 × 10^−5^ m^2^ V^−1^ s^−1^. The much higher mobility in the devices featuring the composite ETL and HTL would presumably improve exciton dissociation and charge transfer to the cathode and anode.^41,42^ Fig. S5† displays steady state PL and time-resolved PL spectra from which we determined qualitatively whether the modified ETL and HTL could transport free carriers more efficiently than could the pristine PC_61_BM ETL and PEDOT:PSS HTL themselves through the perovskite's luminous characteristics. All of the spectra featured an emission peak at 756 nm, the characteristic emission peak of perovskite, with the samples containing the ETL and HTL providing a less intense signal.^43^ From Fig. S5(a),† we find when the Bphen concentration was 0.5 wt%, the PL peak intensity decreased further, but it increased in the case of a Bphen concentration of 0.75 wt%. Based on the ETL containing 0.5 wt% Bphen, the emission when the HTL was doped with MoS_2_ (0.1 wt%) was almost the same as that when it was prepared without MoS_2_. We use bi-exponential decay function fitting to the curves in the Fig. S5(b)† and then obtained average PL decay time (*t*_average_) of 60.6 ns for PEDOT:PSS/perovskite/PC_61_BM and 27.7 ns for PEDOT:PSS (0.1 wt% MoS_2_)/perovskite/PC_61_BM (0.5 wt% Bphen). The sample incorporating the doubly modified transfer layers significantly reduced the PL decay time, implying that photogenerated carriers are efficiently extracted and transferred from the perovskite to the electrode which is consistent with the results from the steady state PL spectra.^44,45^ Typically, traps in the ETL and HTL increase the probability of interfacial recombination of carriers, with a lower degree of interfacial recombination of carriers decreasing the FF of a solar cell device.^46^ Because of the high conductivity of PEDOT:PSS and the high carrier mobility in the perovskite layer, electron transport in the ETL should be the limiting step for charge extraction.^47^ From Table 1, the FFs of the devices prepared with and without MoS_2_ were similar (71.5 and 72%, respectively); thus, the HTL incorporating MoS_2_ improved the efficiency in terms of higher mobility and a greater value of *V*_oc_.

**Fig. 5:**
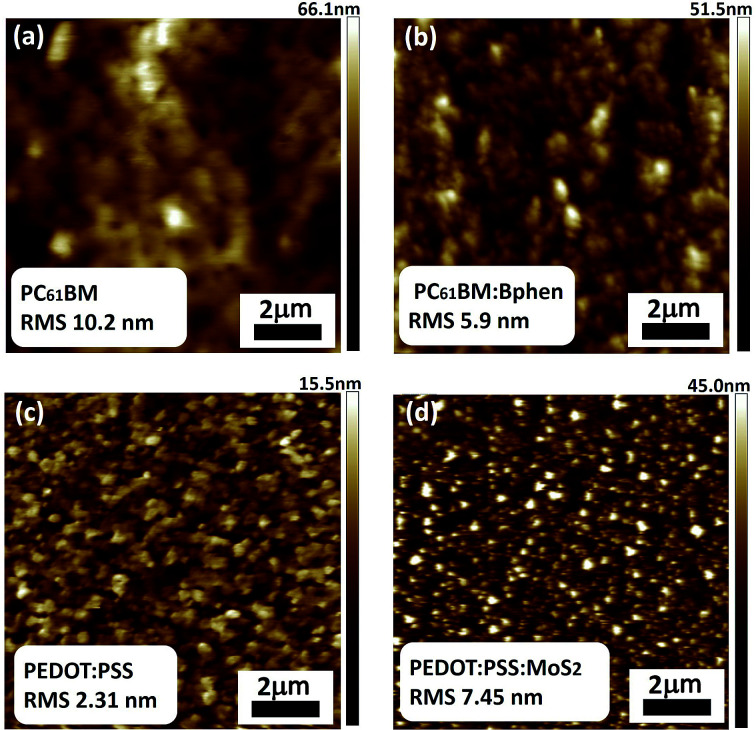
AFM topographic images of films of (a) PC_61_BM, (b) PC_61_BM:Bphen (0.5 wt%), (c) PEODT:PSS, and (d) PEDOT:PSS:MoS_2_ (0.1 wt%).

Fig. 5 presents the topologies of PC_61_BM, PC_61_BM:Bphen (0.5 wt%), PEDOT:PSS, and PEDOT:PSS:MoS_2_ (0.1 wt%), recorded using AFM in tapping mode (scan area: 10 μm × 10 μm). The root-mean-square (RMS) roughness provides a measure of the surface texture, with a smooth film generally having a low value. To minimize contact resistance on the electrode, the contact interface between the perovskite and the electrode should be smooth and compact. After doping Bphen into PC_61_BM, the ETLs had surface characteristics superior to that of the ETL prepared without Bphen, according to the RMS measurements. Fig. 5(b) reveals that the ETL incorporating Bphen at 0.5 wt% had lower RMS roughness (5.9 nm); consistently, the mean FF in the ETL containing 0.5 wt% Bphen was higher than that in the ETLs without Bphen. On the other hand, Fig. 5(d) reveals that the RMS of PEDOT:PSS:MoS_2_ (0.1 wt%) was higher than that of neat PEDOT : PSS because the MoS_2_ sheets are insoluble in water and can only disperse; thus, although the surface was rougher, it was good for hole transmission from the perovskite by offering more fast channels for the holes.^48–50^

**Fig. 6:**
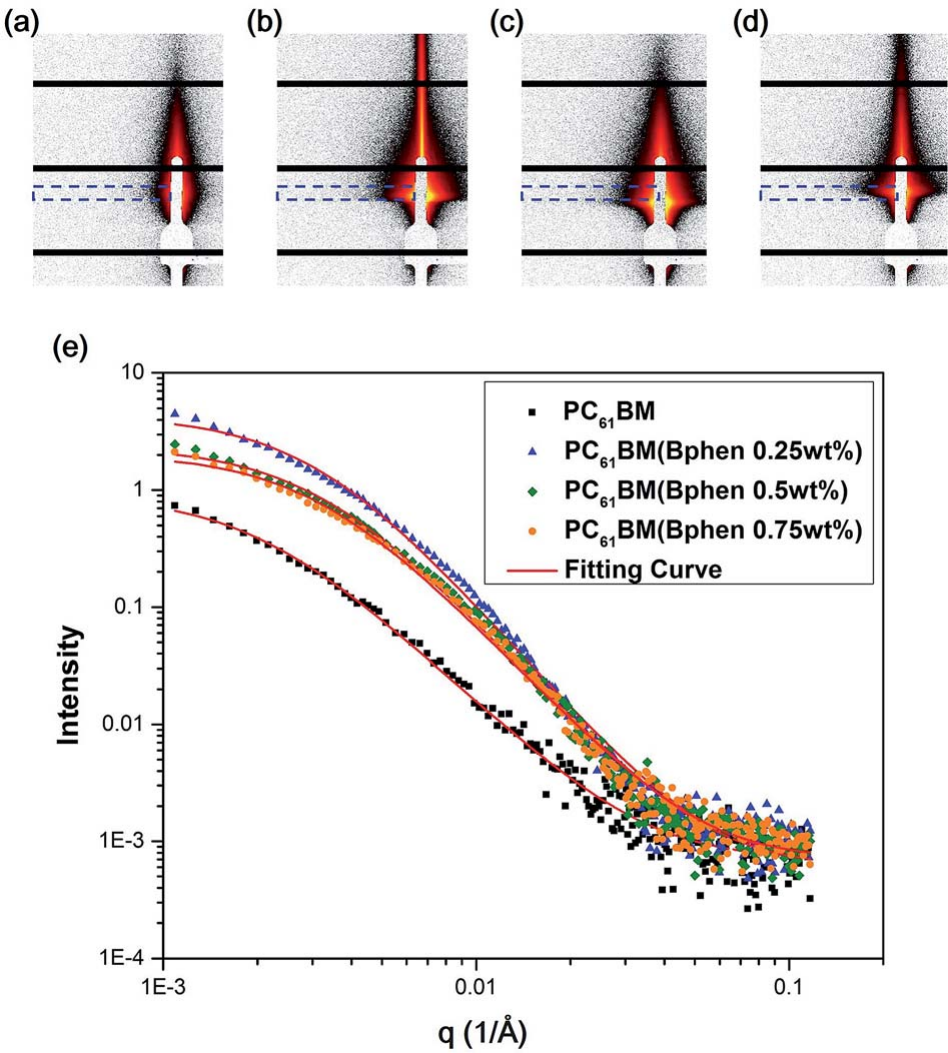
(a–d) 2-D GISAXS patterns of PC_61_BM films containing (a) 0, (b) 0.25, (c) 0.5, and (d) 0.75 wt% Bphen; blue lines represent integral areas. (e) GISAXS profiles of perovskite/PC_61_BM films containing various weight fractions of Bphen. All profiles were fitted using calculated model intensities (solid lines).

Fig. 6(a–d) display 2-D GISAXS patterns of ETLs comprising PC_61_BM:*x*% Bphen (*x* = 0, 0.25, 0.5, 0.75) films spin-cast onto perovskite films on a silicon wafer substrate. We spun the ETLs onto perovskite films on the silicon wafer substrate to prevent the ETLs from forming different morphologies through surface effects. Fig. 6(e) reveals that the one-dimensional (1-D) profiles of the ETLs decreased along the in-plane direction of the 2-D GISAXS patterns; these 1-D GISAXS profiles were deducted from the background of the perovskite on the silicon wafer. We calculated the PC_61_BM cluster sizes in the PC_61_BM:Bphen composite films by fitting the GISAXS scattering intensity [*I*(*q*)]profiles, using eqn (1):

1 *I*(*q*) = *P*(*q*)×*S*(*q*) + *bkg*

2
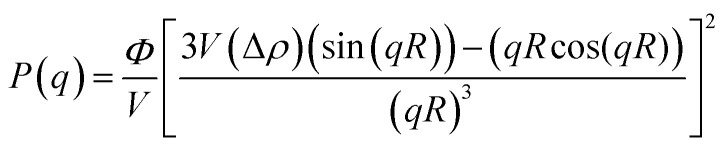
3
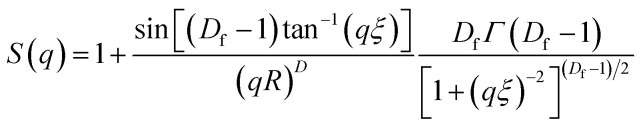


The scattering intensity, *I*(*q*), is determined by the form factor *P*(*q*) of spherical pores as the primary unit [eqn (2)]; *S*(*q*) is the structure factor *S*(*q*), describing the interaction between the primary pores in this fractal-like aggregation network system. In eqn (2), *R* is the mean radius of the spherical pores, *Φ* is the volume fraction, and Δ*ρ* is the difference in the scattering length density (SLD) between spheres and solvent. The value of *S*(*q*) in eqn (3) is related to the correlation length (*ξ*) of the fractal-like network cluster domain formed through aggregation of spherical particles and the fractal dimension (*D*_f_). The domain size of the fractal network can be approximated by the Guinier radius (radius of gyration; *R*_g_ = *ξ* [*D*_f_ (*D*_f_ + 1)/2]^1/2^). Table S2† lists the fitting results for the PC_61_BM:*x* wt% Bphen (*x* = 0, 0.25, 0.5, 0.75) blend films; they have fractal dimensions (*D*_f_) between 2 and 3 and domain sizes (*R*_g_) of 95, 69, 66, and 65 nm, respectively. The ETL prepared from pure PC_61_BM had a large PC_61_BM domain size (95 nm) in the fractal network structure; it decreased to 65–69 nm after incorporating the Bphen small molecules. Although pristine PC_61_BM had a larger domain size, PC_61_BM blended with Bphen had a higher volume fraction, indicating that the electrons had more paths through which they could undergo transfer. When *x* was equal to 0.5, the volume fraction was much larger than that when no Bphen was present, resulting in the highest PCE of 14.8%. The results from the GISAXS characterization of the fractal system were complementary to those from the bright-field TEM images (see Fig. S6†). The dark regions in TEM images represent PC_61_BM cluster dispersions, revealing that aggregation of the PC_61_BM clusters was minimized and that the dispersion of PC_61_BM became more even than that of the neat PC_61_BM when doping with Bphen. Thus, the local morphology observed through TEM may provide very rough evidence of a fractal object at a certain length scale. Therefore, we combined the results of our TEM and GISAXS analyses to establish schematic representations of the PC_61_BM morphologies. Bphen decreased the PC_61_BM cluster size and, accordingly, generated more PC_61_BM aggregation domains to provide more pathways for electron transport. Consequently, the ETLs containing Bphen exhibited good performance in terms of FFs, thereby resulting in PCEs higher than those obtained in the absence of Bphen.

**Fig. 7:**
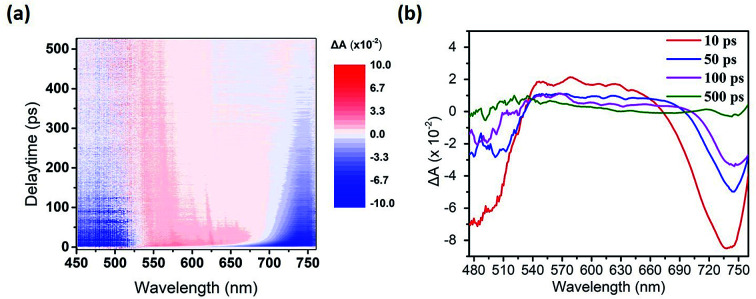
(a) 2-D Plot of the ultrafast transient absorption difference (Δ*A*) on the structure: ITO/PEDOT:PSS:MoS_2_ (0.1 wt%)/CH_3_NH_3_PbI_3−*x*_Cl_*x*_/PC_61_BM:Bphen (0.5 wt%). (b) Transient absorption spectra at various time delays.

We used the pump-probe technique to measure the time- and photon energy-resolved transient absorption difference, Δ*A*(*ω*,*t*), of the control sample (ITO/PEDOT:PSS/CH_3_NH_2_PbI_3−*x*_Cl_*x*_/PC_61_BM) and the sample featuring doubly modified transfer layers [ITO/PEDOT:PSS:MoS_2_ (0.1 wt%)/CH_3_NH_2_PbI_3−*x*_Cl_*x*_/PC_61_BM:Bphen (0.5 wt%)]. Fig. 7(a) presents 2-D plots of the Δ*A* spectra as functions of time and photon energy for the doubly modified transfer layer sample; Fig. S7† presents them for the control sample. The samples were pumped at 400 nm to fill the perovskite conduction band; the resulting absorption dynamics were probed using a white light continuum from 450 to 760 nm. Fig. 7(b) indicates three regions of Δ*A* signals: one negative Δ*A* signal (photoinduced absorption, PIA) at a wavelength of 540–660 nm and two positive Δ*A* signals (photobleached, PB) at wavelengths of approximately 500 and 700 nm, respectively. The broad PIA suggests increased photoexcitation at off-peak wavelengths, due to conduction band saturation.^51^ The two PB signals, PB1 near 700 nm and PB2 near 500 nm, imply blocked optical transitions from the two valence bands excited to the conduction band by the pump pulse.^52^ We chose PB1 to study the photobleaching dynamics, because PB2 is primarily dependent on the presence of impurities rather than bulk phase charge transport.^53^

**Fig. 8:**
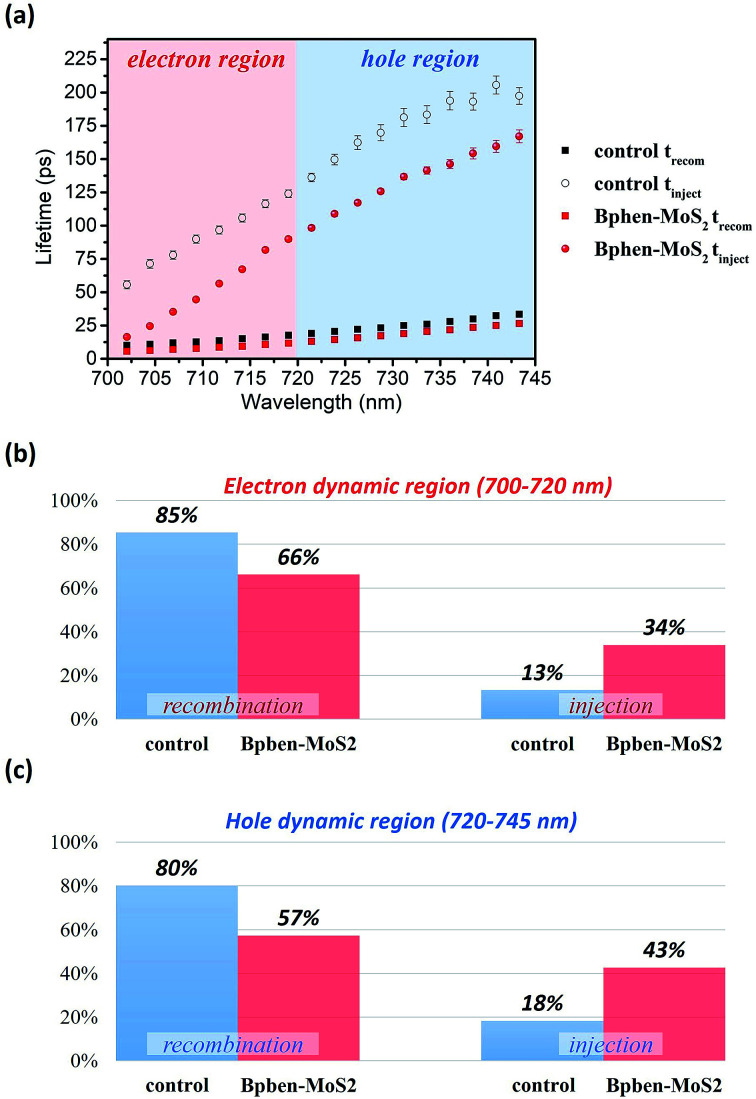
(a) Wavelength-dependent lifetimes extracted from the values of Δ*A*. Average percentages for the decomposed relaxation processes in the two different regions: (b) 700–720 nm for electrons and (c) 720–745 nm for holes.

Fig. 8(a) displays the results of fitting the probe spectrum at wavelengths of 700–745 nm. We extracted amplitudes and lifetimes by fitting the integrated PB peak intensity to a convolution of the excitation Gaussian pulse to the following biexponential function:
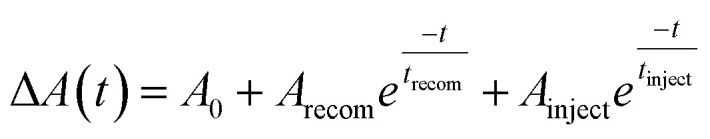
where *t*_recom_ and *t*_inject_ represent to recombination and charge injection lifetimes, respectively; *A*_recom_ and *A*_inject_ represent the contributions from the corresponding components; and *A*_0_ is a constant corresponding to a recombination process having a much longer lifetime. The fast component (*t*_recom_) is related to both charge carrier trapping at the perovskite grain boundaries or defect sites and Auger recombination; the slow component (*t*_inject_) is related to carrier injection from the perovskite to the ETL or HTL. According to previous studies, we used wavelengths of 700–720 nm for the electron dynamics and 720–745 nm for the hole dynamics.^48,50^ Compared with the control sample, the sample featuring the doubly modified transfer layer had shorter time constants in their values of *t*_recom_ and *t*_inject_. Because we modified only the ETL and HTL, and not the perovskite, the decrease in the value of *t*_recom_ was not obvious. In contrast, the value of *t*_inject_ decreased significantly. For the electron region at 710 nm, the values of *t*_inject_ for the control sample and for the sample featuring the doubly modified transfer layers were 89.9 and 44.6 ps, respectively; for the hole region at 735 nm, the values of *t*_inject_ were 183.4 and 141.4 ps, respectively. Thus, for both electrons and holes, the charge injection lifetimes decreased; thus, we confirmed that when incorporating Bphen into PC_61_BM and MoS_2_ into PEDOT:PSS, the probability of recombination at the interface between the perovskite and the transfer layer could decrease, with the shorter lifetime of charge injection potentially increasing the value of *J*_sc_. We calculated the percentage of carriers from the perovskite to the ETL or HTL, using *A*_recom_ and *A*_inject_ to estimate the branching ratio. Fig. 8(b) and (c) present the decomposed components (*A*_recom_, *A*_inject_) in the regions 700–720 nm for electrons and 720–745 nm for holes. We used the expression *A*_inject_/(*A*_recom_ + *A*_inject_ + *A*_0_) to represent the percentage of carrier injection for the process in which carrier transfer occurs from the perovskite boundary to the ETL or HTL.^54^ In these two regions, the percentages of electron and hole injection increased by 21 and 25%, respectively. Thus, there were more effective free carriers, which produced a larger value of *J*_sc_, resulting in the device incorporating the doubly modified transfer layers having the highest value of PCE.

## Conclusions

We have fabricated planar perovskite photovoltaics that incorporate nanocomposite electron transport layer (PC_61_BM:Bphen (0.5 wt%)) and hole transport layer (PEDOT:PSS:MoS_2_ (0.1 wt%)), and demonstrated a corresponding device exhibiting a PCE of 16%—an increase of 57% relative to that (10.2%) for the corresponding planar perovskite device prepared with pristine PC_61_BM as the ETL and pristine PEDOT:PSS as the HTL. The incorporated Bphen and MoS_2_ allowed us to tune the energy level for the composite ETL and HTL, respectively, and increase the carrier mobility. From AFM, we understand the organic molecular Bphen and inorganic molecular MoS_2_ effect the morphology between the transfer layer and electrode. Form grazing-incidence small angle X-ray scattering and transmission electron microscopy analyses, we conclude that the morphological and inner structural properties of the PC_61_BM films incorporating Bphen were superior to those of the pristine PC_61_BM film, forming smaller but more fullerene clusters domains that generated more electron pathways. With transient absorption spectra analyses, we also found that the Bphen and MoS_2_ can improve the carrier lifetime from the perovskite to the transfer layer, and thus enhance the amount percentages of electron injection and hole injection increase by 21% and 35%, resulting in higher values of *J*_sc_ and thus PCE (16%).

## Conflicts of interest

There are no conflicts to declare.

## Supplementary Material

RA-008-C8RA01532E-s001
