# Supplementary material for: Dual nanocomposite carrier transport layers enhance the efficiency of planar perovskite photovoltaics
Source: RSC Adv. 2018 Apr 4;8(23):12526–34. doi: 10.1039/c8ra01532e (PMC9079579; doi:10.1039/c8ra01532e)
Supplement: RA-008-C8RA01532E-s001 [file RA-008-C8RA01532E-s001.pdf]

Electronic Supplementary Material (ESI) for RSC Advances.
This journal is © The Royal Society of Chemistry 2018

Supporting Information

Dual nanocomposite carrier transport layers enhance the efficiency of planar perovskite photovoltaics

Hsi-Kuei Lin[a], Jia-Xing Li[b], Hao-Zheng Wang[a], Yu-Wei Su[c], Kaung-Hsiung Wu[b] and Kung-Hwa Wei[a]*

[a] Department of Materials Science and Engineering, National Chiao Tung University, 300 Hsinchu, Taiwan

[b] Department of Electrophysics, National Chiao Tung University, 300 Hsinchu, Taiwan

[c] Department of Chemical Engineering, Feng Chia University, Taichung 40724, Taiwan

*E-mail: khwei@mail.nctu.edu.tw

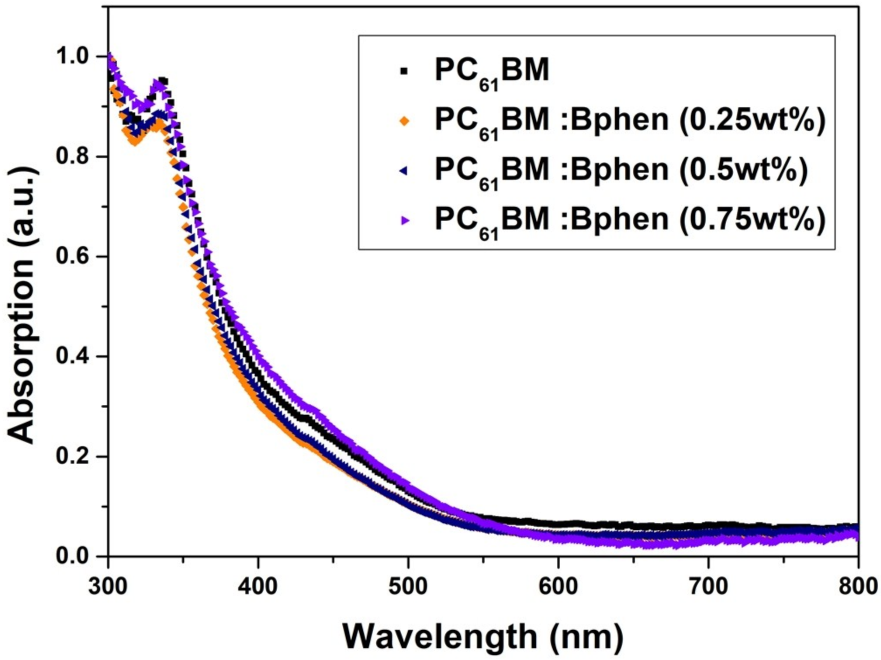

Fig. S1 UV-vis absorption spectra of $PC_{61}BM$ incorporating Bphen at various contents (0, 0.25, 0.375, 0.5 wt%).

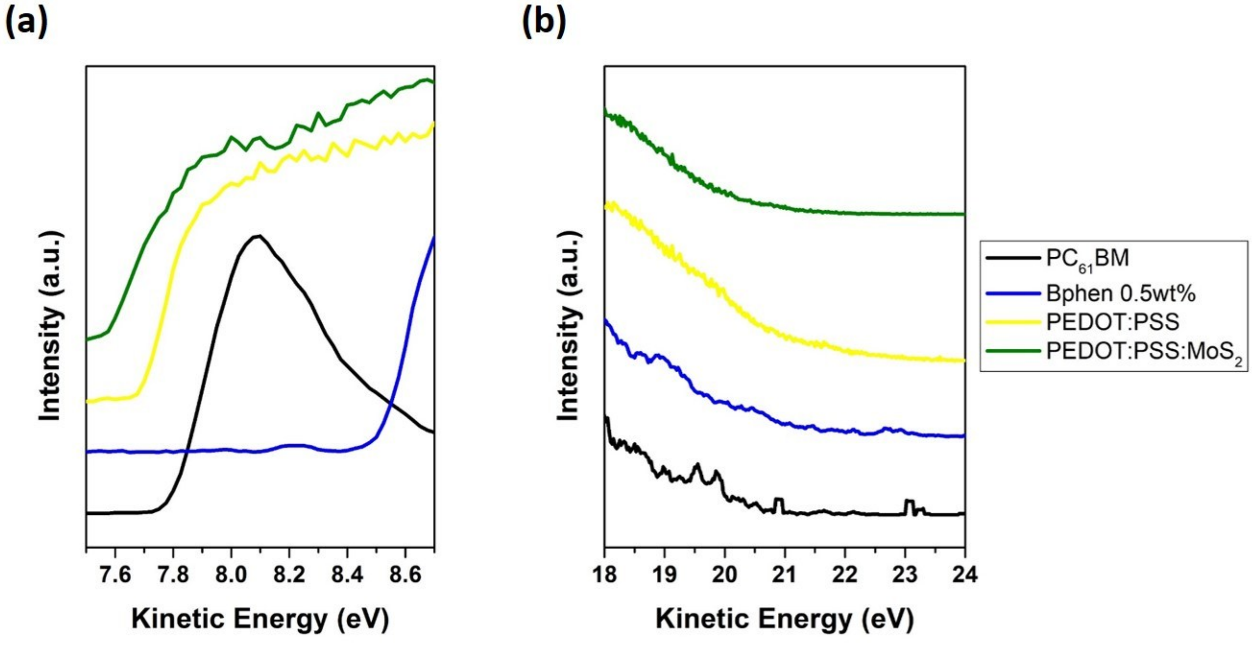

Fig. S2 UPS spectra of $PC_{61}BM$, $PC_{61}BM$:Bphen(0.5wt%), PEDOT:PSS and PEDOT:PSS:MoS2(0.1 wt%) in (a) cutoff energy regions and (b) onset energy regions.

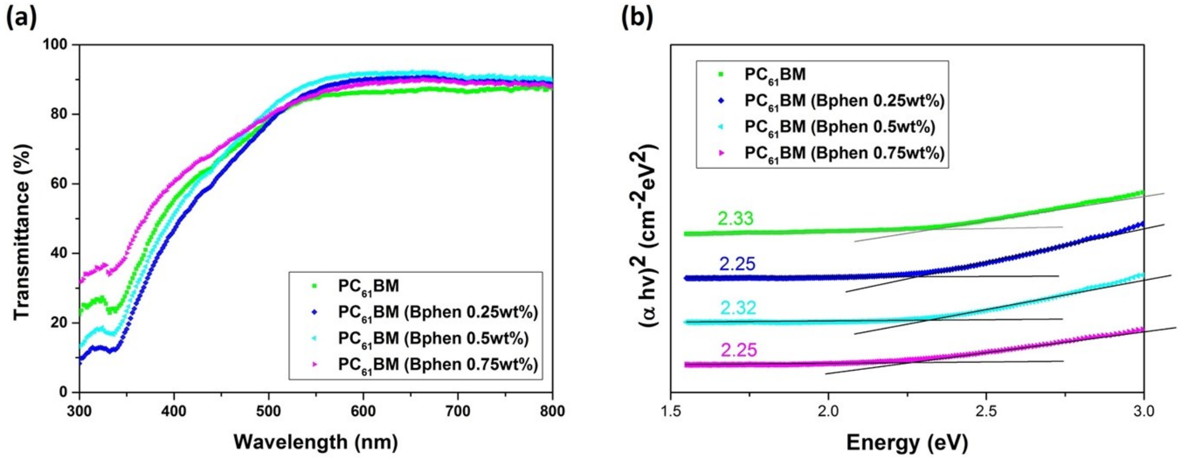

Fig. S3 (a) UV–Vis transmission spectra of films processed using $PC_{61}BM$ incorporating Bphen at various contents (0, 0.25, 0.375, 0.5 wt%). (b) Energy gap in $PC_{61}BM$ incorporating Bphen at various contents (0, 0.25, 0.375, 0.5 wt%) by calculating through a Tauc plot.

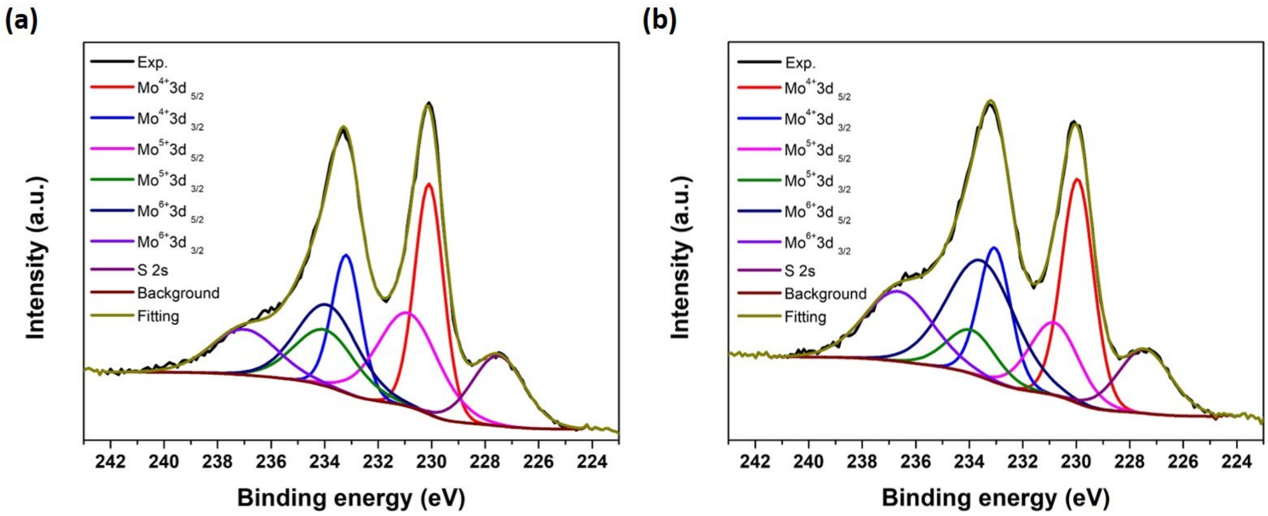

(c)

XPS area ratio (%)	Mo^{4+}	Mo^{5+}	Mo^{6+}	S 2s
MoS_2	33.7	29.2	25.5	11.6
MoS_2 UV treatmanet	34.5	17.3	38.1	10.1

Fig. S4 XPS narrow-scan spectra (Mo 3d) of perovskite films prepared (a) with and (b) without UV-ozone treatment; (c) XPS area ratios of chemical states of Mo species.

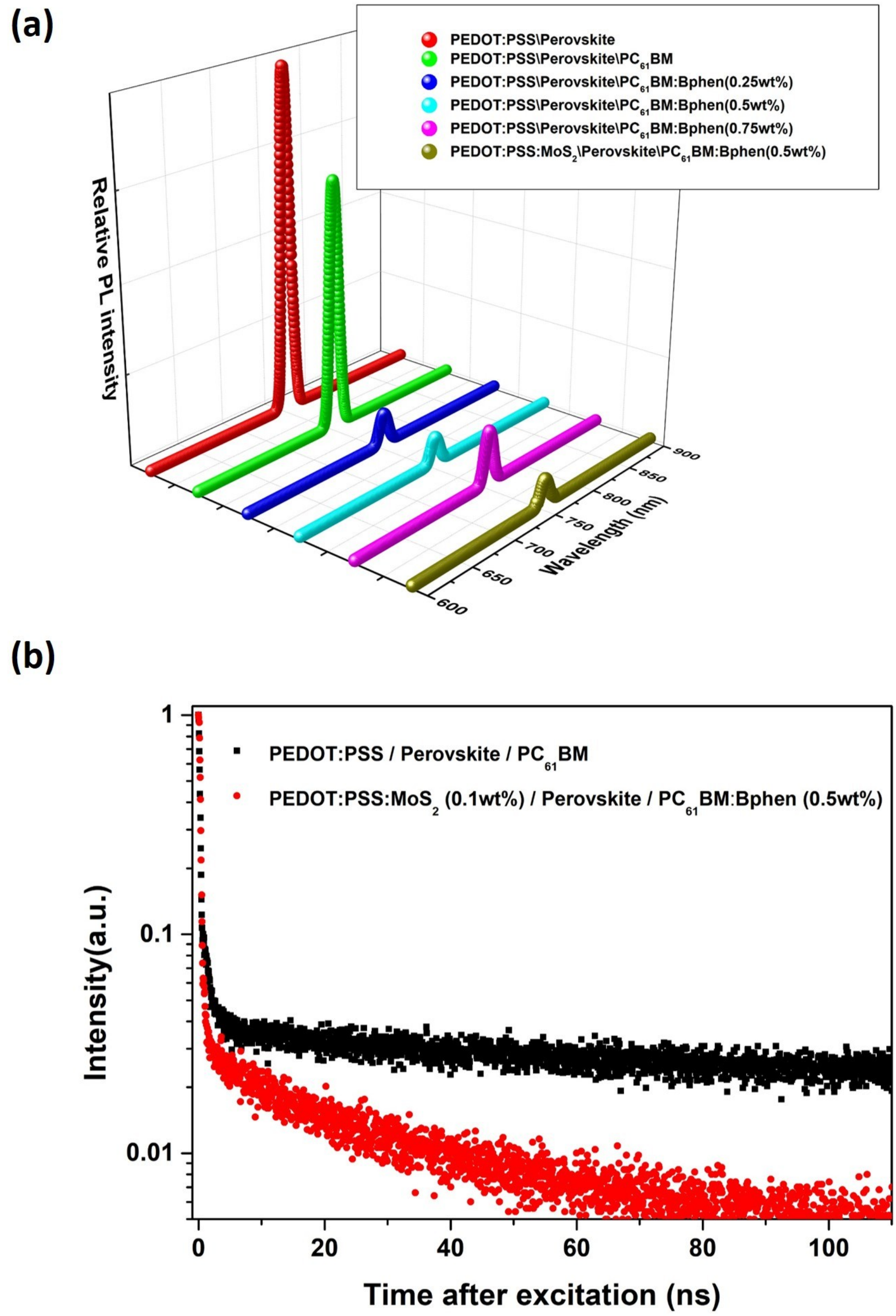

Fig. S5 (a) steady state PL spectra of films processed using PEDOT:PSS/perovskite, PEDOT:PSS/perovskite/$PC_{61}BM$ incorporating Bphen at various contents (0, 0.25, 0.5, 0.75 wt%) and PEDOT:PSSMoS_2 (0.1wt%)/Perovskite/$PC_{61}BM$:Bphen (0.5wt%). (b) time-resolved photoluminescence (TRPL) spectra of PEDOT:PSS/perovskite/$PC_{61}BM$ and PEDOT:PSSMoS_2 (0.1wt%)/Perovskite/$PC_{61}BM$:Bphen (0.5wt%).

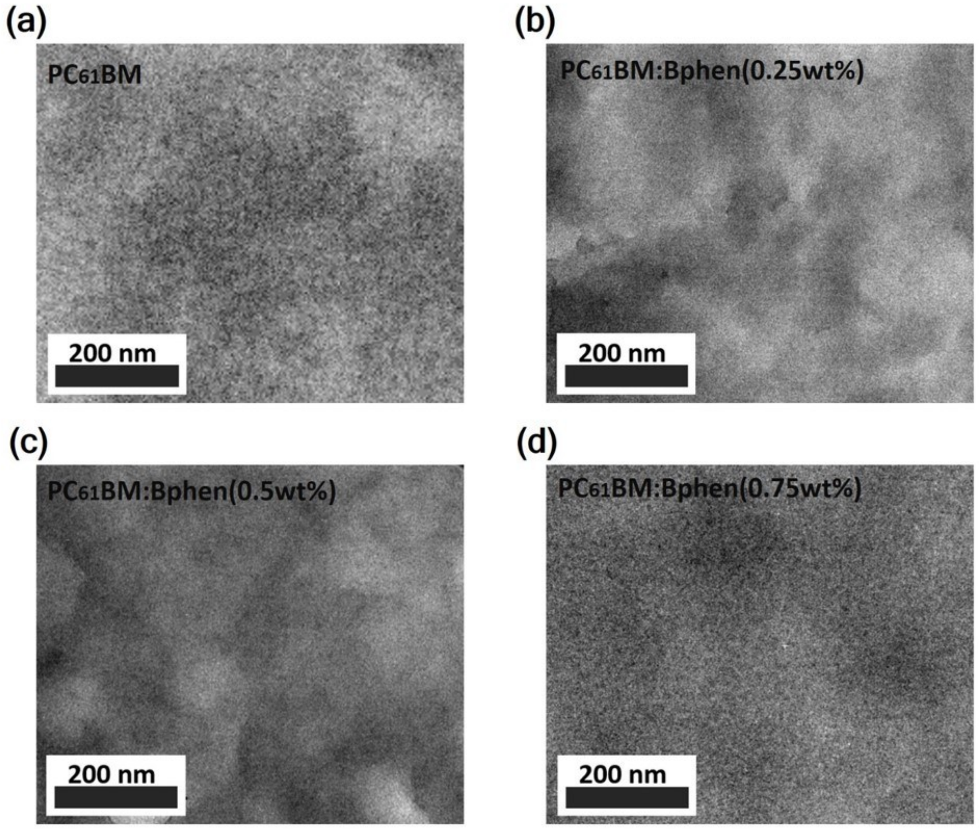

Fig. S6 TEM images of PC61BM films incorporating Bphen at various contents (0.25, 0.5, 0.75 wt%).

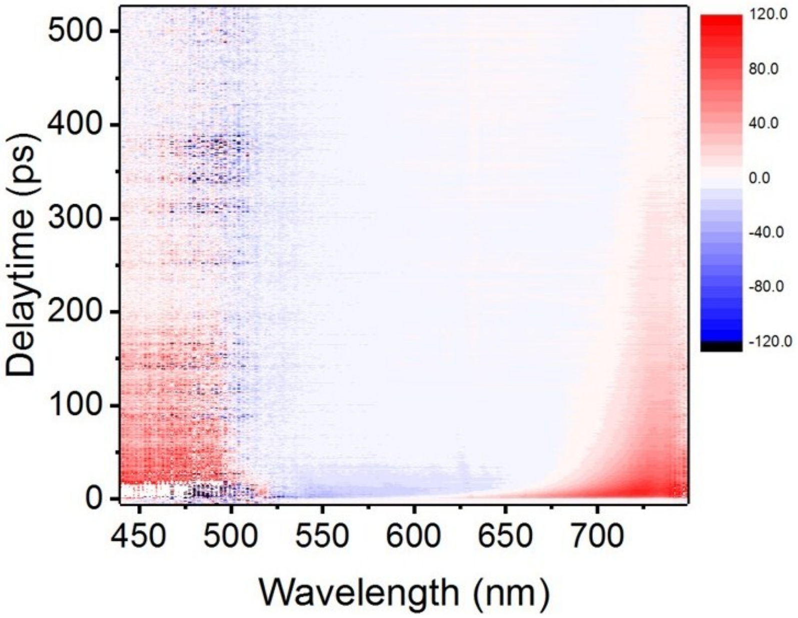

Fig. S7 2-D plots of ultrafast transient absorption difference Δ*A* on ITO/PEDOT:PSS/CH3NH$_3$PbI$_{3-x}$Cl$_x$/PC$_{61}$BM.

Table S2 Fitting parameters of 1-D GISAXS profiles of the $PC_{61}BM$:x wt% Bphene (x = 0, 0.25, 0.5 and 0.75)

Parameter	$PC_{61}BM$	$PC_{61}BM$: x wt% Bphen		
		x = 0.25	x = 0.5	x = 0.75
Φ: volume fraction	3E-4	9.12E-4	8.2E-3	7.9E-3
R: mean radius (Å)	5.7	5.5	5.1	4.8
Df: fractal dimension	2.39	2.67	2.56	2.55
ξ: correlation length(Å)	472	312	308	304
SLD sphere ($Å^{-2}$)	4.1E-6	4.1E-6	4.10E-6	4.1E-6
SLD solvent ($Å^{-2}$)	4.7E-6	4.7E-6	4.70E-6	4.7E-6
bkg (cm^{-1} sr^{-1}).	7E-4	5E-4	5E-4	7E-4
Domain size: Rg (nm)	95	69	66	65

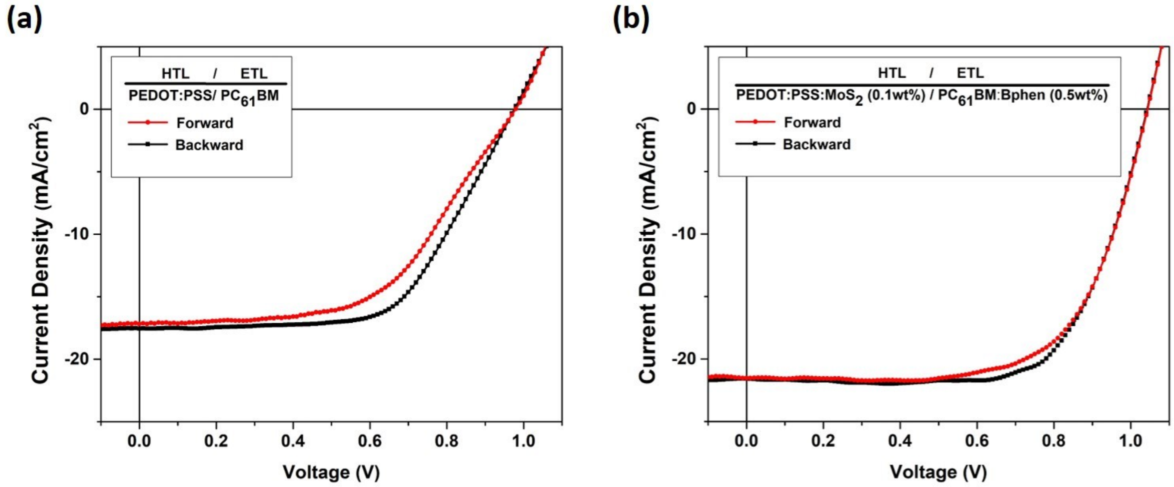

Fig. S8 Current density–voltage (J–V) characteristic curves of the perovskite solar cells at forward scan and backward scan for control device and doubly modified with carrier transport layers device.
